# Toll-like receptors in cardiac hypertrophy

**DOI:** 10.3389/fcvm.2023.1143583

**Published:** 2023-04-11

**Authors:** Yanan Zhang, Jimin Wu, Erdan Dong, Zhanli Wang, Han Xiao

**Affiliations:** ^1^ Inner Mongolia Key Laboratory of Disease-Related Biomarkers, The Second Affiliated Hospital, Baotou Medical College, Baotou, China; ^2^ Department of Cardiology and Institute of Vascular Medicine, Peking University Third Hospital, Beijing, China; ^3^ NHC Key Laboratory of Cardiovascular Molecular Biology and Regulatory Peptides, Peking University Third Hospital, Beijing, China; ^4^ Key Laboratory of Molecular Cardiovascular Science, Ministry of Education, Beijing, China; ^5^ Beijing Key Laboratory of Cardiovascular Receptors Research, Peking University Third Hospital, Beijing, China; ^6^ Research Unit of Medical Science Research Management/Basic and Clinical Research of Metabolic Cardiovascular Diseases, Chinese Academy of Medical Sciences, Beijing, China; ^7^ Department of Clinical Laboratory, Shanghai University of Medicine & Health Sciences Affiliated Zhoupu Hospital, Shanghai, China

**Keywords:** cardiac hypertrophy, toll-like receptor, innate immune, inflammation, signaling pathway

## Abstract

Toll-like receptors (TLRs) are a family of pattern recognition receptors (PRRs) that can identify pathogen-associated molecular patterns (PAMPs) and damage-associated molecular patterns (DAMPs). TLRs play an important role in the innate immune response, leading to acute and chronic inflammation. Cardiac hypertrophy, an important cardiac remodeling phenotype during cardiovascular disease, contributes to the development of heart failure. In previous decades, many studies have reported that TLR-mediated inflammation was involved in the induction of myocardium hypertrophic remodeling, suggesting that targeting TLR signaling might be an effective strategy against pathological cardiac hypertrophy. Thus, it is necessary to study the mechanisms underlying TLR functions in cardiac hypertrophy. In this review, we summarized key findings of TLR signaling in cardiac hypertrophy.

## 1. Introduction

**Figure 1:**
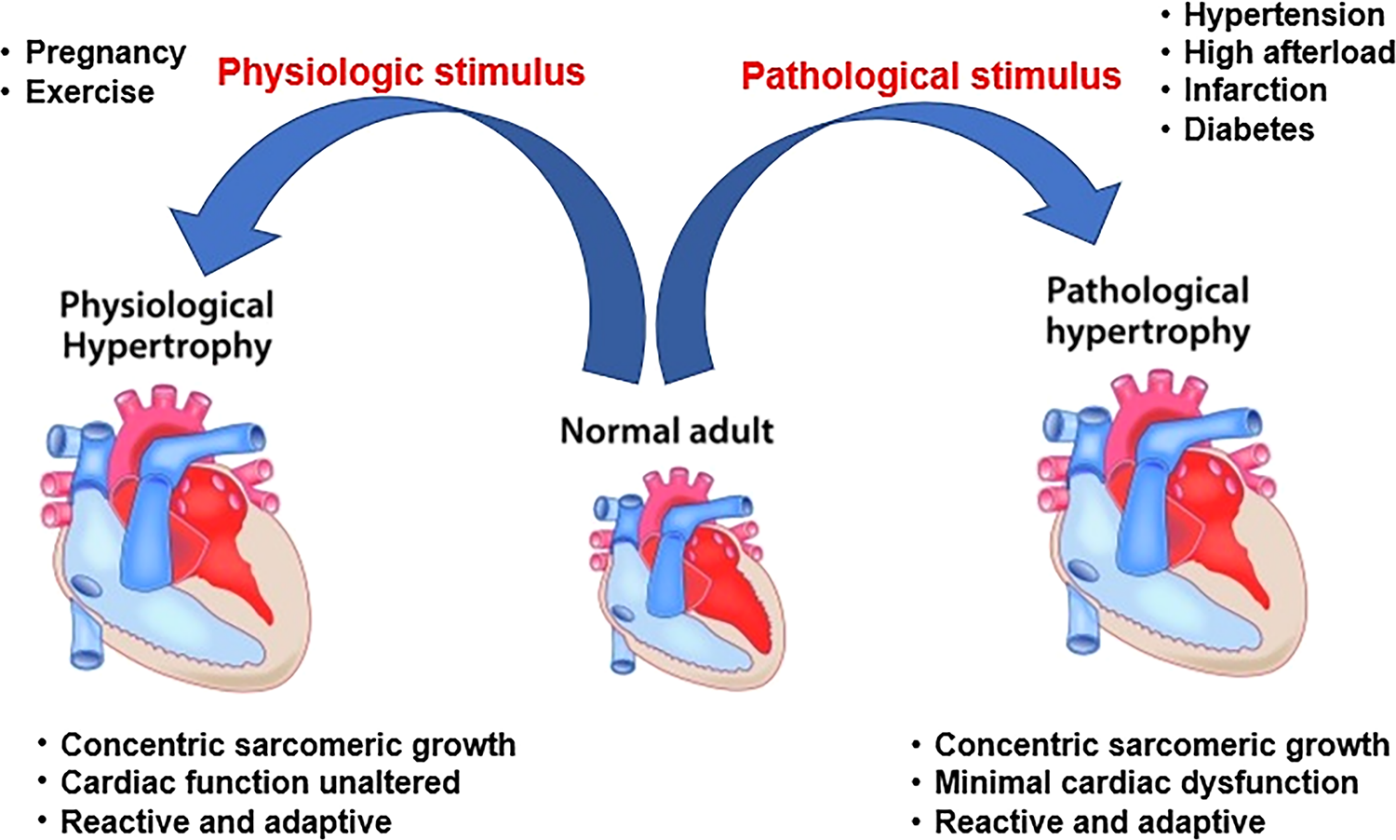
Phenotypes of physiological and pathological cardiac hypertrophy.

Cardiac hypertrophy, characterized by an enlargement of cardiomyocyte size, is initially an adaptive response to various stimuli (Figure 1) (1). Physiological cardiac hypertrophy occurs in response to pregnancy and exercise to preserve or improve heart function without cardiac fibrosis (2). In contrast, pathological cardiac hypertrophy accompanying myocardial dysfunction and fibrosis is the cardiac response to chronic stressful conditions, such as hypertension and valvular disease (3). Pathological cardiac hypertrophy plays a causal role in the progression of heart failure. Pathological hypertrophy is associated with increased interstitial fibrosis, cell death, and cardiac dysfunction, as well as increased production of proinflammatory cytokines (3, 4). Inflammation is a characteristic feature of pathological cardiac hypertrophy (5). Toll-like receptors (TLRs), as innate immune receptors, are key factors in cardiovascular diseases (6). Insights into the precise function of TLR-mediated cardiac inflammatory signaling will aid in developing novel therapies for pathological cardiac hypertrophy.

## 2. TLRs and downstream adaptors

TLRs have been first discovered in *Drosophila melanogaster*, playing a pivotal role in embryonic development and dorsal-ventral polarity (7, 8). The researchers have further found that the function of TLRs is related to innate and adaptive immunity (9). To date, 13 and 10 TLRs have been identified in mice and humans, respectively. Each TLR recognizes distinct microbial components. For example, a heterodimer of TLR2/1 or TLR2/6 recognizes lipoproteins, TLR3 responds to double-stranded RNA (dsRNA), TLR4 senses lipopolysaccharide (LPS), TLR5 binds to bacterial flagellin, TLR7/8 respond to single-stranded viral RNA (ssRNA), and TLR9 recognizes bacterial DNA containing unmethylated CpG motifs (10–15).

**Figure 2:**
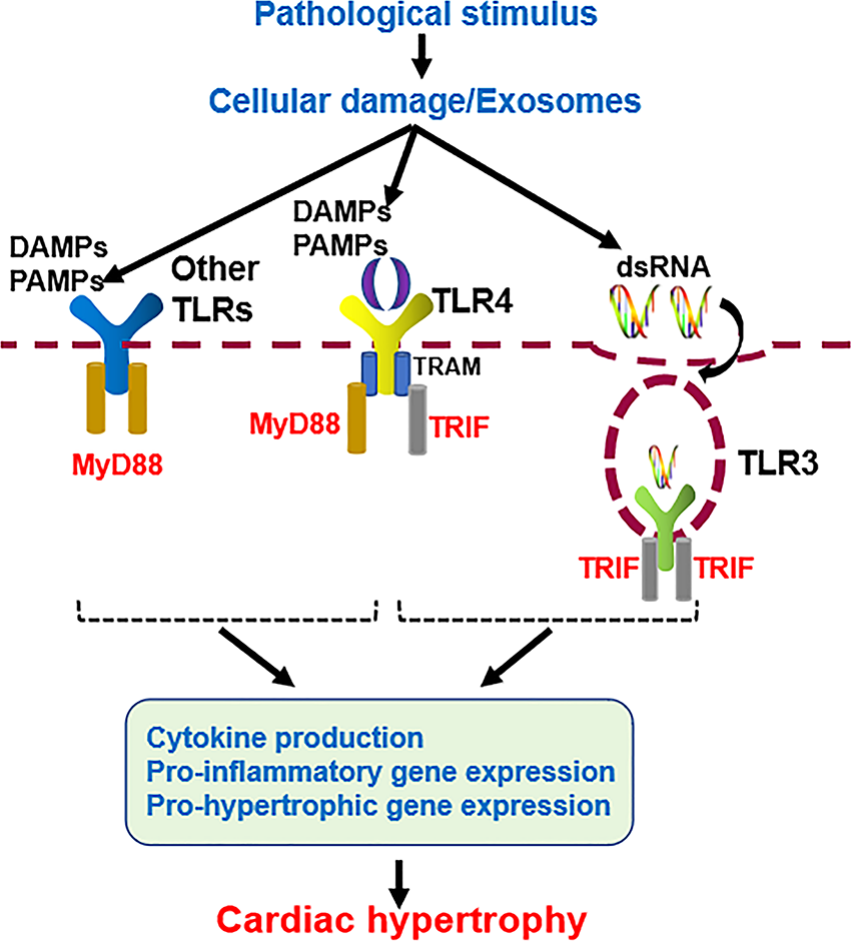
Cardiac hypertrophy mediated by MyD88-dependent and independent TLRs signaling pathways.

TLR family members usually dimerize themselves and recruit adaptor molecules with the same Toll- interleukin-1 (IL-1) receptor (TIR) domain to transmit signals. TLRs signals can be divided into myeloid differentiation factor 88 (MyD88)-dependent and MyD88-independent pathways (Figure 2). Except for TLR3, the signals of all TLR family members are conducted through the MyD88-dependent pathway, which induces the expression of proinflammatory cytokines, chemokines, and other inflammation-related molecules by activating nuclear factor-κB (NF-κB) and other transcription factors (8, 16). TLR3 signals through the MyD88-independent pathway, which includes another adaptor molecule, TIR domain-containing adaptor-inducing interferon-β (TRIF), also known as the TRIF-dependent pathway (17). TLR4 is the only TLR that triggers both MyD88- and TRIF-dependent pathways (18, 19). MyD88 and TRIF are TLR adaptor molecules, and other adaptor molecules include TIR domain-containing adaptor protein (TIRAP) and TRIF-related adaptor molecule (TRAM) (20–23).

Numerous specific protein serine/threonine kinases participate in TLR signaling, such as IL-1 receptor-associated kinases (IRAKs), the transforming growth factor-β-activated kinase 1 (TAK-1), and the IκB kinase (IKK) complex. On the other hand, protein serine/threonine phosphatases, phospho-protein phosphatases (PPPs), metal-dependent protein phosphatases (PPMs), and aspartate-based phosphatases counterbalance and limit TLR signaling (24).

Different TLRs recognize specific ligands with distinct PAMPs and DAMPs, whereas all TLRs signals activate NF-κB. The excessive activation of TLR receptor signaling can also lead to autoimmune and inflammatory diseases (25). Consequently, different TLRs in a given pathological state may yield different outcomes that define the phenotype of tissue injury and organ damage.

## 3. Inflammation is involved in the pathogenesis of cardiac hypertrophy

Common pathophysiological mechanisms associated with cardiac hypertrophy include oxidative stress (26, 27), renin-angiotensin-aldosterone system (RAAS) (28), nervous system-activated sympathetic activity (29), pressure overload (30), and inflammation (31, 32). Inflammation is the pathological basis of myocardial hypertrophy (32). Other diseases such as hypertension (33) and ischemic injury (34) also provoke inflammatory responses, leading to cardiac hypertrophy. TLRs are widely expressed in many cells in the heart, and activating TLR-mediated inflammation signaling pathways promotes immune cell migration to the heart and cardiac fibroblast differentiation (35, 36). Therefore, TLR-induced inflammatory signaling is significant in the development of cardiac hypertrophy.

Importantly, immune cells play a pivotal role in the inflammatory response, but their cardioprotective or cardiodestructive effects differ after pressure overload. Particularly, neutrophils, dendritic cells (DCs), and mast cells demonstrate destructive functions in animal models, whereas eosinophils and natural killer T cells display cardioprotective activities (37). For example, S100 calcium-binding protein A8/A9 complex (S100a8/a9), an initial proinflammatory factor produced by neutrophils, activates the NF-κB pathway in angiotensin II (Ang II)-induced cardiac fibrosis and hypertrophy (38). Besides, TLR stimulation and DC infiltration are factors contributing to heart failure (39). Cardiac macrophages, which are highly plastic cells, are divided into two types of macrophages, including proinflammatory (M1) and anti-inflammatory (M2) phenotypes. M1 macrophages are associated with chronic inflammation, and M2 macrophages produce IL-10 and TGF-β1, which are related to tissue repair and fibrotic properties (33, 40). TLR4 activator LPS stimulates macrophages to produce mir-155 that promotes cardiac inflammation, followed by cardiac fibrosis, apoptosis, and hypertrophy (41). Overall, inflammatory cells infiltrate the heart by activating intracellular inflammatory signaling pathways, eventually contributing to cardiac hypertrophy and heart failure.

## 4. Toll-like receptors are associated with cardiac hypertrophy

TLRs are major components of the innate immune system that elicit cytokine and chemokine production primarily by activating the proinflammatory transcription factor NF-κB (18). Herein, we review several important TLRs associated with cardiac hypertrophy (Table 1).

**Table 1:** Summary of various factors that can interact with specific TLRs, leading to cardiac hypertrophy and cardiac remodeling.

Factors	Animal Model	Downstream signaling	Phenotype	*In vivo* or *in vitro*	Reference
HSP70	TAC/Dox	TLR2/NF-κB	Inflammation, cardiac hypertrophy, heart failure	*In vivo* and *in vitro*	(42, 43)
IL-1β	Trypanosoma cruzi	TLR2/NF-κB	Cardiac hypertrophy	*In vitro*	(44)
SNO-MLP	Phenylephrine/AngII	TLR3/RIP3/NLRP3	Cardiac hypertrophy, heart failure	*In vivo*	(45)
Palmitic acid	Obesity	TLR4/c-Src/EGFR	Cardiovascular diseases	*In vitro*	(46)
LPS	LPS	TLR4/MyD88/CaMKII	LPS/MI-induced hypertrophic and inflammatory	*In vivo*	(47)
MCP-1	Ang II	TLR4	Cardiac hypertrophy and dysfunction	*In vivo*	(48)
RBP4	Ang II	TLR4/MyD88	Insulin resistance and cardiac hypertrophy	*In vivo*	(49)
MD2	Ang II	TLR-4/MyD88/NF-κB	Cardiac inflammation and remodeling	*In vivo* and *in vitro*	(50)
fibrinogen	TAB	TLR4/MyD88/NF-κB	Cardiac hypertrophy	*In vivo*	(51)
STAT3	Ang II	IL-6/gp130/JAK2/STAT3	Cardiac dysfunction and remodeling	*In vivo*	(52)
Flagellin	AB	TLR5	Interstitial cardiac fibrosis and dysfunction	*In vivo*	(53)
Resiquimod	SLE	TLR7/8	Autoimmune-mediated dilated cardiomyopathy	*In vivo*	(41)
T. cruzi	Chagas’ disease	TLR7/STAT3	Cardiomegaly and myocardial failure	*In vitro*	(54)
miR-101	PE/TAC	XIST/miR-101/TLR2	Cardiac hypertrophy	*In vivo* and *in vitro*	(55)
dsRNA	Ang II	TLR3/TRIF	Cardiac hypertrophy and hypertension	*In vivo*	(56)
LncRNA (CTPB1-AS2)	Ang II	SP1/CTPB1-AS2/TLR4	Cardiac hypertrophy	*In vivo*	(57)
ssRNA	Enteroviral RNA	TLR8/MyD88	Enterovirus-associated DCM	*In vivo*	(58)
Mitochondrial DNA	DNase II-deficient heart	TLR9	Chronic inflammation and heart failure	*In vivo*	(59)

ACTA-1, α-actin; AAC, abdominal aortic constriction; CaMKII, calcium/calmodulin-dependent protein kinase II; DOX, doxorubicin; HSP70, heat shock protein-70; LPS, lipopolysaccharide; MCP-1, monocyte chemoattractant protein 1; MD2, myeloid differential protein-2; PE, phosphatidylethanolamine; RBP4, retinol-binding protein 4; SNO-MLP, S-nitrosylation of muscle LIM protein; STAT3, signal transducer and activator of transcription 3; SLE, systemic lupus erythematosus; TAC, transverse aortic constriction; TRIF, toll-interleukin-1 receptor-domain-containing adapter-inducing interferon-β.

### 4.1. TLR2

TLR2 in complex with TLR1 or TLR6 is essential for recognizing bacterial lipoproteins and lipopeptides. After recognizing their ligands, the TLRs form stable TLR1-TLR2 or TLR2-TLR6 complexes (60). TLR2 activation has been associated with cardiovascular diseases (61, 62). Ye et al. have shown that TLR2 mediates cardiac hypertrophy and inflammation in Ang-II-treated mice through the TLR2/MyD88/NF-κB signaling pathway. Ang II significantly increased the level of the TLR2-MyD88 complex rather than that of TLR2 or MyD88 protein (63). Additionally, TLR2 activation upregulates NF-κB and inflammatory factors, such as IL-1β, which can induce cardiomyocyte hypertrophy and fibroblast and vascular endothelial cell proliferation (44). TLR2/NF-κB/IL-1β signaling is essential for activating the IGF-1/PI3K/Akt pathway and leads to adaptive cardiac hypertrophy during pressure overload (64). Besides, TLR2 is involved in renal ischemia/reperfusion (I/R)-induced cardiac hypertrophy by regulating the systemic inflammatory profile and NF-κB activation (65). A recent study has suggested that lncRNA X-inactive specific transcripts (XIST) could induce cardiac hypertrophy by targeting miR-101 and increasing TLR2 levels (55). Besides, studies have demonstrated that heat shock proteins (HSPs), such as HSP60 and HSP70, induced cardiac hypertrophy by activating NF-κB through the TLR2/MyD88-dependent pathway in Dox-induced animal models (42, 43). In contrast, HSP25 protects the heart from Dox-induced cardiotoxicity by antagonizing the binding of Dox to the TLR2 receptor (66). TLR2 deficiency in cardiac cells prevents Ang II-induced cardiac remodeling, inflammation, and dysfunction by reducing the formation of TLR2-MyD88 complexes (67). Obesity has been studied as an activator of DAMPs, which use the TLR2 signaling pathway to increase cytokine expression in heart tissue (68). Although TLR2 has been shown to induce cardiac hypertrophy, several studies have suggested that TLR2 is required for cardiac protection. TLR2-deficient mice have shown short-term advantages after myocardial I/R but promoted left ventricular dilation in the long term with reduced collagen and decorin density in the infarct scar (69). TLR2 stimulation also protects the heart from exaggerated autoimmunity in experimental autoimmune myocarditis by promoting regulatory DCs formation, which limits autoreactive T-cell responses (70). Therefore, the role of TLR2 in cardiac hypertrophy is destructive or protective, depending on the etiology and disease stage.

### 4.2. TLR3

TLR3 is located in the endoplasmic reticulum. Upon stimulation with dsRNA, TLR3 moves to the endosomes, where TLR3 is phosphorylated by Bruton's tyrosine kinase (BTK) and phosphorylated IRF3, triggering its downstream signaling (71, 72). Its adaptor protein for the dsRNA-induced signaling pathway is not MyD88 but TRIF (73, 74). TRIF also recruits additional proteins necessary for downstream signaling, including receptor-interacting protein 1 (RIP1), TNF receptor-associated factor 3 (TRAF3), nucleosome assembly protein 1 (NAP1), and TBK1. The TLR3/TRIF pathway then activates NF-κB and IFN regulatory factor 3 (IRF-3) (56). TLR3 deficiency in mice with Coxsackie virus B3 (CVB3) infection increases viral replication during the acute period of myocarditis. TLR3 deficiency also increases the level of cytokines related to T helper (Th) 2 response, such as IL-4, IL-10, IL-13, and TGF-β. IL-4 deficiency in mice improves heart function during acute CVB3 myocarditis, suggesting that TLR3 prevents myocarditis by reducing viral replication and IL-4 levels in the heart (75). S-nitrosylation of muscle LIM protein (MLP) induces TLR3-mediated RIP3 and nucleotide-binding oligomerization domain-like receptor pyrin domain containing 3 (NLRP3) inflammasome activation, thereby promoting the development of myocardial hypertrophy (45). Although Ang II activates both MyD88 and TRIF pathways, only the TRIF pathway is required to mediate hypertension and cardiac hypertrophy (76). A recent study has found that both TLR4-TRIF and TLR3-TRIF pathways mediate Ang II-induced cardiac hypertrophy, whereby only the TLR3-TRIF pathway is required for Ang II-induced hypertension (77).

### 4.3. TLR4

PAMPs and DAMPs act as exogenous or endogenous ligands for TLR4, respectively. Its co-receptor myeloid differentiation protein 2 (MD2) recognizes LPS and binds TLR4, followed by the activation of the TLR4 signaling pathway (78). Additionally, hyperthyroidism, enteroviral replication, and lifestyle-related diseases directly compromise the myocardial structure and lead to inflammation through TLR4 and downstream activation of the NLRP3 inflammasome or NF-κB-dependent pathways (16, 79, 80). For example, postnatal growth restriction (PNGR) and hyperoxia cause intestinal dysbiosis that activates pulmonary hypertension and, subsequently, promotes right ventricular hypertrophy *via* the TLR4/NF-κB/IL-1β pathway (81). Besides, TLR4 activation increases oxidative stress and activates MCP-1 expression, resulting in cardiac hypertrophy in Ang II-induced hypertension (48). TLR4 is the only member of the TLRs family that simultaneously activates intracellular signal transduction through two different signaling pathways, the MyD88-dependent and MyD88-independent pathways.

In the MyD88-dependent pathway, LPS binds to LPS-binding protein (LBP), and this complex then binds with CD14, transferring LPS to TLR4 and its co-receptor MD2 through hydrogen bonding on Arg-90, Glu-92, and Asp-100 (50, 82–84). Inside the cells, this CD14/TLR4/MD2 compound interacts with adaptor TIRAP, inducing IL receptor-associated kinase (IRAK) phosphorylation, MyD88 separation, and TRAF6 combination. Then, TRAF6 can activate NF-κB through TGF-β activated kinase 1 (TAK1) and MAPKs, such as JNK, extracellular-signal-regulated kinase (ERK), and p38 kinase, through mitogen-activated protein kinase ERKA 6 (MKK6), which, in turn, activates AP-1, leading to the expression of proinflammatory cytokines (51, 85–87). Several different inflammatory cytokines, including TNF-α, IL-6, and IL-1β, are induced through this signaling pathway (88). For example, retinol-binding protein 4 (RBP4) contributes to insulin resistance and heart failure by activating the TLR4/MyD88 signaling pathway (49).

Other MyD88-dependent pathways include the TLR4/MyD88/CaMK II, TLR4/MyD88/PI3K/Akt, and TLR4/MyD88/MAPK pathways, showing that TLR4/MyD88 downstream is more complicated in regulating cardiac hypertrophy. CaMK II belongs to serine/threonine kinases and plays an important role in cardiac structure remodeling and electrical activity (89). MyD88 leads to CaMK II oxidation and is essential for adverse cardiac hypertrophy and inflammation during myocardial infarction (47). The TLR4/MyD88/PI3K/Akt pathway has both adverse and protective effects on cardiac hypertrophy, probably due to the different PI3K isoforms. PI3K p110*γ* activates maladaptive cardiac hypertrophy, whereas PI3K p110α induces adaptive cardiac hypertrophy (90).

The MyD88-independent pathway is also named the TRIF-dependent pathway. IKK*ε* and TBK1 are molecules downstream of TRIF, which activate NF-κB and IRF3, respectively (91, 92). NF-κB releases IκB from the binding complex, leading to NF-κB translocation from cytosol to the nucleus.

Interestingly, some molecules induce cardiac hypertrophy by multiple pathways. Nucleotide-binding oligomerization domain-2 (NOD2)-knockdown in mice increases cardiac hypertrophy and fibrosis by upregulating multiple pathways, including the TLR4/NF-κB, TLR4/MAPK, and TGF-β/Smad pathways (93). Besides, Ang II activates STAT3, which interacts with TLR4 and increases IL-6, and, in turn, promotes the second STAT3 activation, leading to an upregulated expression of genes for cardiac hypertrophy through the IL-6/glycoprotein 130 (gp130)/Janus-family tyrosine kinases 2 (JAK2) pathway (52).

### 4.4. TLR5

TLR5, a transmembrane protein, is highly expressed in immune cells, cardiomyocytes, and vascular endothelial cells. TLR5 triggers inflammatory responses and promotes cardiac hypertrophy, and the deficiency of TLR5 in mice attenuates the cardiac hypertrophy and dysfunction induced by pressure overload (53). TLR5 directly interacts with spleen tyrosine kinase and activates NADPH oxidase, stimulating the p38 MAP kinase pathway in DOX-induced cardiotoxicity (94).

### 4.5. TLR7/8

TLR8 mediates the antiviral response by recognizing ssRNA. TLR8 is associated with the immune response to enteroviral replication and may be involved in enterovirus-associated dilated cardiomyopathy (58). Additionally, both *T. cruzi* trypomastigotes (extracellular form) and amastigotes (intracellular form) induce cardiomyocyte apoptosis *via* TLR7 signaling to activate transcription factor STAT3, which then upregulates apoptotic gene *BAX* and downregulates anti-apoptotic gene *BCL-2* (54). Furthermore, TLR7/8 agonist resiquimod causes myocarditis and dilated cardiomyopathy, mimicking the cardiac damage induced by systemic autoimmune diseases, such as systemic lupus erythematosus and rheumatoid arthritis. Furthermore, the cardiac damage may be due to the systemic increase in inflammation or the direct autoimmune response toward the heart (95).

### 4.6. TLR9

TLR9, a receptor for unmethylated CpG-DNA, bacterial DNA, viral DNA, and fungi, was first cloned and identified in 2,000 (96–99). When TLR9 is activated by binding with its ligands, it can induce a TLR9-mediated immune response, such as an antiviral response, and the production of type I IFN through plasmacytoid DCs (100). TLR9 induces NF-κB *via* the MyD88-dependent pathway, where CD82 acts as an important regulator of TLR9-mediated signaling in cancer, infectious diseases, and autoimmune diseases (101). Furthermore, TLR9 triggers innate and adaptive immune responses against pathogens, such as *Brucella, Streptococcus pneumoniae, Helicobacter*, mouse cytomegalovirus (MCMV), herpes simplex virus (HSV) types 1 and 2, and adenovirus (102–108). Bacterial DNA could mediate neutrophil signaling by TLR9-independent and MyD88-dependent pathways (109). Mitochondrial DNA escapes from cell autophagy and leads to TLR9-mediated inflammatory responses in cardiomyocytes, followed by myocarditis and dilated cardiomyopathy (59). Inhibiting TLR9/NF-κB-mediated sterile inflammation also improves pressure overload-induced right ventricular dysfunction (110). However, TLR9 also mediates the cardiac protection of oligonucleotides or peptides. Synthetic oligonucleotides (ODNs), such as CpG-ODN C274 and 1668-thioate, attenuate ISO (isoproterenol) or I/R-induced cardiac hypertrophy by activating TLR9-mediated PI3K/AKT signaling (111, 112). Wang et al. have demonstrated that cathelicidin-related antimicrobial peptide (CRAMP) inhibited the cardiac hypertrophic response by activating the IGFR1/PI3K/AKT pathway and ameliorated cardiac oxidative stress by activating the TLR9/AMPK pathway in cardiomyocytes. TLR9 is required for the anti-oxidative effect of mCRAMP, as demonstrated by using TLR9-knockout mice. Additionally, TLR9 knockout partly reverses the antihypertrophic effect of mCRAMP, suggesting that TLR9 also contributes to protecting cardiomyocytes from hypertrophy induced by pressure overload (113).

## 5. Potential therapeutic approaches in cardiac hypertrophy

Several promising drugs and technologies have been developed to attenuate TLR-mediated inflammatory response and reverse cardiac hypertrophy (114). Thus, TLRs and TLR signaling medications might be potential treatment approaches in cardiac hypertrophy.

An alternative therapeutic strategy is blocking TLR upstream molecules to diminish inflammation and attenuate cardiac hypertrophy. For example, some protein molecules, such as modified citrus pectin (a specific inhibitor of galectin-3), cardiac transmembrane BAX inhibitor motif containing 1 (TMBIM1), HMGB1, EGFR, human mesenchymal stem cells, and erythropoietin (EPO), in the heart reverse pressure overload-induced cardiac hypertrophy by blocking the TLR4 signaling pathway (109, 115–117). Inhibiting some nucleic acid molecules, such as lncRNA CTPB1-AS2, lncRNA NEAT1 and miR-93, can ameliorate cardiac hypertrophy by downregulating TLR4 signaling (57–119). Silencing of protein molecule-like fatty acid-binding protein 4 protects against LPS-induced cardiomyocyte hypertrophy and apoptosis by inhibiting the TLR4/NF-κB pathway (120). Besides, CaMKII*δ*B silencing prevents cardiac hypertrophy independent of an inflammatory response by inhibiting the complement system and TLR2/4 NF-kB signaling (121).

In addition, TLR inhibitors can decrease cardiac hypertrophy. Some chemical compounds, such as choline and eritoran, ameliorate cardiac hypertrophy by inhibiting TLR4, which decreases inflammatory cytokines, such as IL-1β and IL-6, and increases anti-inflammatory cytokines, such as IL-10 (122, 123). Other chemical compounds, such as Triad3A (a ubiquitin E3 ligase), TAK-242, and lipopolysaccharide from *Rhodobacter sphaeroides* (LPS-RS), have been reported to negatively regulate the NF-κB activation pathway *via* the inhibition of TLR4/TLR9 or TLR4 and subsequently inhibit cardiac disease (124–127). Additionally, Ang II-induced microglia activation and oxidative stress are linked to TLR4 activation in the paraventricular nucleus (128). Inhibiting TLR4 within the paraventricular nucleus (PVN, an important cardioregulatory center in the brain) attenuates blood pressure and inflammation (129). Calcitriol infusion in the PVN ameliorates hypertensive responses and cardiac hypertrophy by decreasing TLR4-associated inflammation (130). Recombinant human relaxin (RLX) and bioactive peptides attenuated cardiac hypertrophy, inflammation, and fibrosis and appeared to involve the inhibition of TLR4 (131, 132). Interestingly, studies have reported that silencing *TLR4* gene through siRNA prevents the development of diabetic cardiomyopathy in streptozotocin-induced type 1 diabetes (133).

Some TLR/MyD88 signaling inhibitors also ameliorate cardiac hypertrophy. Receptor-interacting serine/threonine-protein kinase 2 (RIP2) deficiency ameliorates cardiac hypertrophy through multiple signaling pathways that reduce TLR4/MyD88/NF-κB activation and MAPKs phosphorylation (134). In contrast, some molecules and compounds negatively regulate cardiac hypertrophy by suppressing TLR4/MyD88 signaling, which includes protein molecules such as MD1 and anti-HSP70 antibody and compounds such as Ang II type 1 receptor (AT1-R) antagonist and liver × receptors agonist (135–138). Besides, long-term oral atazanavir attenuates myocardial infarction-induced cardiac fibrosis by targeting the HMGB1/TLR9 signaling pathway (139). Pharmacologic inhibition of the MyD88 inhibitor, ST2825/IMG2005, protects against pathologic cardiac remodeling (140, 141). Moreover, other types of cardiovascular drugs, such as telmisartan, magnesium isoglycyrrhizinate (MgIG), dimethyl fumarate (DMF), and statins, including atorvastatin and simvastatin, effectively suppress the TLR-4/NF-κB signaling pathway and protect against cardiac remolding in pressure overload, chronic intermittent hypoxia, and LPS-induced conditions (46, 142–145). Traditional Chinese Medicine drugs, such as Shenqi Yangxin decoction (SQYXD), *Lycium barbarum* polysaccharide (LBP), arbutin, Astragaloside IV (AsIV), and Dangshen Erling decoction (DSELD) have been shown to attenuate cardiac hypertrophy by targeting the high mobility group box 1 (HMGB1)/receptor for advanced glycation end products (RAGE) and TLR4/NF-κB signaling pathway (146–150). A recent report showed that double overexpression of miR-19a and miR-20a (dOex-mIRs) in human induced pluripotent stem cell (iPS)-derived mesenchymal stem cells (MSCs) effectively preserves the left ventricular function in dilated cardiomyopathy through, at least in part, regulating TLR4/MAL/MyD88 signaling pathway (151). Nevertheless, more clinical trials and reliable measurements regarding therapeutic approaches targeting TLR signaling pathways are needed. The factors that inhibit TLR signaling-mediated cardiac hypertrophy and cardiac remodeling are listed in Table 2.

**Table 2:** Summary of various inhibitors that can interact with TLR signaling and ameliorate cardiac hypertrophy and cardiac remodeling.

Factors	Animal model	Downstream signaling	Effect	*In vivo* or *in vitro*	Reference
miR-93	Ang II	TLR4/PI3K/Akt/mTOR	Ameliorate cardiac hypertrophy	*In vitro*	(118)
mCRAMP	Ang II	TLR9/AMPKa	Completely ameliorate cardiac oxidative stress and partly ameliorate cardiac hypertrophy	*In vivo*	(113)
S100a8/a9	Ang II	TLR4/NF-κB	Prevent inflammatory cell infiltration, perivascular and interstitial fibrosis, and hypertrophy	*In vitro*	(38)
Telmisartan	Ang II	TLR4/MyD88/NF-κB	Attenuate mean arterial pressure, cardiac hypertrophy, and inflammation	*In vivo*	(136)
Triad3A	Ang II	TLR4 and TLR9/NF-κB	Ameliorate cardiac hypertrophy	*In vivo* and *in vitro*	(125)
MgIG	Isoproterenol	TLR4/NF-κB (p65)	Ameliorate myocardial fibrosis	*In vivo*	(143)
DMF	Isoproterenol	TLR4/MyD88/*p*-ERK1/2	Ameliorate cardiac hypertrophy	*In vivo*	(144)
Arbutin	Isoproterenol	TLR4/NF-κB	Ameliorate cardiac hypertrophy	*In vivo*	(149)
LPS-RS	Isoproterenol	TLR4/MyD88	Reduce cardiac redox imbalance, mitochondrial dysfunction, and cardiac hypertrophy	*In vivo*	(127)
HSP25	Dox	TLR2/NF-κB	Prevent cardiac hypertrophy	*In vitro*	(66)
SQYXD	Dox	TLR4/NF-κB	Ameliorate cardiac hypertrophy	*In vivo*	(147)
TAK-242 (TLR4 inhibitor)	Aldosterone	TLR4	Inhibits hypertension, cardiac and renal fibrosis, and epithelial-mesenchymal transition	*In vivo*	(126)
dsRNA	Coxsackievirus B3	TLR3/IL-4	Prevent myocarditis and DCM	*In vivo*	(75)
Eritoran	TAC	TLR4/IL-1β,IL-6	Ameliorate cardiac hypertrophy	*In vivo*	(123)
1668-thioate	TAC	TLR9	Reduce cardiac growth and fibrosis and delay loss of cardiac function	*In vivo*	(112)
NOD2	AB	TLR4/MAPKs/NF-κB/TGFβ/Smad	Attenuate cardiac hypertrophy and fibrosis	*In vivo*	(93)
RIP2	AB	TLR4/MyD88/NF-κB/MAPKs	Ameliorate cardiac hypertrophy, inflammation, and fibrosis	*In vivo*	(134)
TMBIM1	AB	Tumor susceptibility gene 101/TLR4/Lysosome	Ameliorate cardiac hypertrophy and heart failure	*In vivo*	(116)
Choline	Spontaneously hypertensive	TLR4	Improve vagal activity, hypertension, and cardiac hypertrophy	*In vivo*	(122)
*Lactobacillus reuteri* GMNL-263	Diabetes mellitus	TLR-4/NF-κB	Reduce diabetes-induced cardiomyopathy.	*In vivo*	(153)
MD1	High-fat diet	TLR4/MyD88/CaMKII	Ameliorate cardiac hypertrophy, fibrosis, and dysfunction	*In vitro*	(135)

AAC, abdominal aortic constriction; AB, aortic banding; DOX, doxorubicin; DMF, dimethyl fumarate; E6446, TLR9 inhibitor; HSP25, heat shock protein 25; HSP60, heat shoch protein 60; LPS-RS, lipopolysaccharide from the photosynthetic bacterium *Rhodobacter sphaeroides*; MCP-1, monocyte chemoattractant protein-1; MgIG, magnesium isoglycyrrhizinate; MD1, myeloid differentiation protein 1; NOD2, nucleotide-binding oligomerization domain-2; PAB, pulmonary artery banding; RIP2, receptor-interacting serine/threonine-protein kinase 2; SHR, spontaneously hypertensive rats; SHR, spontaneously hypertensive Wistar rats; S100a8/a9, extracellular heterodimeric proteins; SQYXD, Shenqi Yangxin decoction; Triad3A, ubiquitin E3 ligase; TAK-242, TLR4 inhibitor; TMBIM1, transmembrane BAX inhibitor motif containing 1.

Moreover, rather than directly targeting the TLR signaling pathway, some indirect strategies may provide additional therapeutic benefits for cardiovascular diseases. Caloric restriction is an effective therapeutic approach in the treatment of diabetes and associated cardiomyopathy by inhibition of TLR2 and TLR4 (152). Besides, probiotics *Lactobacillus reuteri* GMNL-263, *Bifidobacterium breve* CECT7263 (BFM), *Lactobacillus fermentum* CECT5716 (LC40), and *L. coryniformis* CECT5711 (K8) plus *L. gasseri* CECT5714 (LC9) (1:1), prevent dysbiosis, endothelial dysfunction, endotoxemia, and high blood pressure and ameliorate cardiac hypertrophy *via* the downregulation of their indirect target TLR4 (153–155). Apart from these, renal denervation and repetitive hyperthermia (RHT) attenuate the development of cardiac hypertrophy, at least in part by inhibiting TLR4 expression (19, 156).

## 6. Conclusion and future perspectives

Increased inflammatory factors and cytokines are clearly associated with cardiac hypertrophy and TLRs. In this review, we summarized comprehensive information about TLRs, such as TLR2, TLR3, TLR4, TLR5, TLR7/8, and TLR9, which are closely related to cardiac hypertrophy. TLRs interact with their ligands and co-receptors to induce the expression of numerous inflammatory factors and inflammatory cell infiltration in the heart, leading to cardiac hypertrophy and heart failure through various inflammatory signaling pathways. Reviewing the interaction between TLRs and inflammation in cardiac hypertrophy may be a research direction for the treatment of cardiovascular diseases and other inflammatory-related diseases. However, the link between TLRs and cardiac hypertrophy has not been fully explored. For example, little has been reported about the role of TLR7/8 and TLR9 in cardiac hypertrophy, especially how they mediate inflammatory signaling pathways and heart diseases. Additionally, the TLR family not only regulates inflammation but is also one of the essential mediators of the innate immune response. It is worth noting that the excessive activation of TLRs can lead to chronic inflammation and autoimmune diseases, while TLR defects can lead to cancer and allergies (157, 158). Therefore, the TLR family might play a variety of different roles in cardiovascular diseases. Still, it may need a deeper exploration of the TLR signaling pathway related to cardiac hypertrophy.

The engagement of different TLR ligands leads to unique cytokine production (159). It is likely that cross-talk within various TLR pathways is highly complex and contains many unknowns (160). Although there are many challenges in developing drugs and balancing TLR signaling, in consideration of molecular targeting therapy against TLRs and signaling molecules might be a promising approach in clinical treatment, many research centers and pharmaceutical companies are expending extensive efforts to develop TLRs modulators. Some of the TLR-based agonistic and antagonistic agents have shown to be efficacious in preclinical models and have now entered clinical trials (161, 162). Overall, these interesting findings encouraged us to set a further goal to understand the detailed mechanism of TLR-mediated inflammatory responses and cardiac hypertrophy and identify the potential targets of therapeutic interventions through TLRs' downstream and upstream signaling pathways.
